# Effects of hemodialysis treatment on saliva flow rate and saliva composition during in-center maintenance dialysis: a cross-sectional study

**DOI:** 10.1080/0886022X.2020.1857769

**Published:** 2020-12-17

**Authors:** I-Chen Yu, Chieh-Yu Liu, Ji-Tseng Fang

**Affiliations:** a Department of Nursing, Chang Gung University of Science and Technology, Taiwan; b Department of Nephrology, Chang Gung Memorial Hospital in Linkou, Tao-Yuan, Taiwan; c Department of Speech Language Pathology and Audiology, College of Nursing, National Taipei University of Nursing and Health Sciences, Taipei, Taiwan; d School of Medicine, College of Medicine, Chang Gung University, Tao-Yuan, Taiwan

**Keywords:** Hemodialysis, saliva components, saliva flow rate, unstimulated whole saliva

## Abstract

**Aim:**

To analyze changes in saliva flow rate and clinical measures from unstimulated whole saliva (UWS) among patients undergoing hemodialysis for end-stage kidney disease (ESKD).

**Background:**

Chronic hemodialysis causes changes in blood chemistry as well as dry mouth, due to removal of excess fluids. UWS is used to examine saliva flow rate as an indicator of mouth dryness. Whether UWS can be used to measure changes in clinical variables following hemodialysis has not been explored.

**Design:**

A cross-sectional quantitative study.

**Methods:**

Patients with ESKD were recruited by purposive sampling (*n* = 100) between 1 January and 30 June 2015 from a hospital in northern Taiwan. UWS was collected 1-hour pre-dialysis (T1), mid-dialysis (T2), and 1-hour post-dialysis (T3). Saliva flow rate and clinical variables were analyzed.

**Results:**

Saliva flow rate increased significantly from T1 to T3 (Wald χ^2^ = 10.40, *p* < .01). Changes in saliva from T1 to T3 included decreases in blood urea nitrogen and creatinine (Wald χ^2^ = 97.12, *p* < .001 and Wald χ^2^ = 36.98, *p* < .001, respectively). The pH and osmolality also decreased (*p* < .001 and *p* < .01, respectively). Changes in electrolytes included decreases in potassium and calcium (Wald χ^2^ = 6.71, *p* < .05 and Wald χ^2^ = 17.64, *p* < .01, respectively) and increases in chloride (Wald χ^2^ = 17.64, *p* < .001).

**Conclusion:**

Our findings demonstrated saliva flow rate and several saliva components were altered during hemodialysis. The total volume of saliva secretion increased following dialysis, which can reduce xerostomia. Therefore, medical personnel could provide interventions of relieving dry mouth symptoms and increasing saliva flow rate before hemodialysis treatment.

## Introduction

The prevalence of end-stage kidney disease (ESKD) in Taiwan was reported to be the highest in the world in 2017 by the United States Renal Data System (USRDS); prevalence of treatment, per million population (PMP), was 3,392 PMP, and the incidence was 493 PMP [1]. Nephritis, nephrotic syndrome, and renal disease are among the top 10 leading causes of death in Taiwan and treatments for ESKD contributes a significant financial burden to expenditures for Taiwan’s National Health Insurance System. Worldwide, hemodialysis (HD) is the most common approach for treatment of ESKD [1] and this also holds true for Taiwan, with 83,808 individuals in treatment at the end of 2016 [2].

Patients with ESKD undergo chronic hemodialysis and often suffer from dry mouth. Chronic hemodialysis has additional side effects due to the changes in blood volume that occur over the course of treatment: blood pressure fluctuations, which can lead to interdialytic hypertension; alterations in electrolytes, such as sodium; and fluctuations in biochemicals, such as albumin, blood urea nitrogen (BUN) and creatine (Cr); and weight gain [3]. Therefore, a critical component for optimizing outcomes of hemodialysis is fluid control, which can also influence saliva flow rate.

Saliva flow rate measurement, which includes the measurement of stimulated saliva flow rate and unstimulated saliva flow rate, is a crucial objective indicator for dry mouth diagnosis. Measurement of stimulated saliva flow rate involves having the patient chew on a piece of chemically inert resin or transparent gum at 45 chews/min for 5 min, collecting their secreted saliva, and then calculating the mean weight of saliva secretion [4]. By contrast, measurement of unstimulated saliva flow rate involves measuring the participant’s secreted saliva during 5–10 min of rest without receiving any form of stimulation [5].

Saliva comprises numerous substances. In addition to water and mucin, saliva contains blood urea nitrogen (BUN), creatinine (Cr), and various electrolytes. Relevant research has indicated that the concentrations of biochemical substances in saliva and blood are equally correlated to each other [6]. The saliva secretion volume under normal physiological functioning should be approximately 0.5–1 L/day [7]. A survey conducted in Taiwan indicated that the unstimulated saliva flow rate of hemodialysis patients is approximately 0.06–0.07 mL/min [8]; approximately 66.4% of such patients have experienced dry mouth [9]. Ship et al. determined that unstimulated saliva flow rate below 0.1–0.2 mL/min indicates salivary gland hypofunction (SGH) [10]. Using salivary scintigraphy, Kao et al. found hemodialysis patients who experienced dry mouth had decreased salivary gland functioning [11]. A possible reason underlying the occurrence of dry mouth in dialysis patients is metabolic wastes, such as high concentration BUN and Cr, damaging the salivary gland; this in turn affects the saliva secretion function of the gland and causes the sensation of mouth dryness.

The combined effect of high concentrations of BUN and Cr, removal of water, and changes in electrolytes during the process of hemodialysis also lead to osmolality changes [12,13]. Osmolality changes cause redistribution of cellular water content and decreased blood flow to the kidneys resulting in metabolic changes in the renin–angiotensin system, and stimulation of osmolality receptors, which also cause dry mouth [14]. Additionally, changes in osmolality and electrolytes, such as sodium and potassium, are crucial factors that can cause mouth dryness [15]. The causes of decreased saliva flow may be medications, diseases, or radiotherapy. For example, anticholinergics inhibit saliva secretion. However, patients’ saliva secretion returns to normal after they stop using said medication. Similarly, the continued use of adrenergic agonist antagonists such as clondine, guanfacine and methyldopa decreases saliva secretion [16–18]. Some diseases or treatments result in decreased saliva flow. For instance, Sjögren’s syndrome and patients with head and neck cancer receiving radiotherapy experience decreased saliva flow [5,16]. Sex, age, and smoking habits also affect mouth dryness among patients on hemodialysis [15,19]. Hemodialysis patients often increase their fluid intake to offset dry mouth, which can cause interdialytic weight gain; every 1% increase over a 5% weight gain increases mortality risk by 67.5% [20].

The variables of saliva flow rate, and serum levels of BUN, Cr and electrolytes are all effected by treatment with hemodialysis. By examining saliva changes during the dialysis process, one can better understand the symptom of mouth dryness among patients on hemodialysis. Therefore, the aim of this study was to examine unstimulated saliva flow rates as well as BUN, Cr, and electrolyte levels in saliva samples, and to investigate the effects of hemodialysis treatment on these variables during hemodialysis and 1 h after the completion of hemodialysis. Our findings could serve as an objective basis for future nursing interventions for patients experiencing dry mouth due to chronic hemodialysis.

## Materials and methods

### Design

This cross-sectional study used repeated measures of saliva samples collected 1 h prior to dialysis (T1), midway through dialysis (T2), and 1 h after completion of dialysis (T3).

### Participants

Participants were recruited by purposive sampling of patients with ESKD from a hemodialysis center of a regional teaching hospital in the Taoyuan area of Taiwan. Patients who met the following inclusion criteria were invited to participate in the study: between the ages of 20 and 80 years; receiving hemodialysis on a regular basis; had experienced mouth dryness in the past 4 weeks; fully conscious; able to spit out saliva; and able to communicate in Mandarin, Taiwanese, or written text. Exclusion criteria were as follows: presence of head and neck cancer and receiving radiotherapy; Sjögren’s syndrome; or had taken any of the following medications within the past week: diuretics, tricyclic antidepressants, anticholinergics, antihistamines, or antianginals.

This study was reviewed and approved by the Institutional Review Board (IRB) of the study hospital (IRB Landseed 14-025-B1). A researcher identified all eligible participants. The first author described the purpose and design of the study and assured them they had the right to withdraw at any time, without any loss of benefits to which they were otherwise entitled. All patients who agreed to participate provided written informed consent prior to data collection. Participants then filled out a survey questionnaire (described below) and a time was arranged that was determined convenient for each participant for collection of hemodialysis -related data. Participants were instructed not to engage in oral activities, such as smoking, eating, drinking, or teeth brushing, for 1 h prior to arrival at the clinic for dialysis.

### Measurements

#### Demographic and clinical characteristics

A survey questionnaire collected data regarding demographic characteristics of participants included gender, age, and health behaviors. Clinical characteristics included smoking (yes/no), duration of hemodialysis treatment and comorbidities. Clinical data collected prior to dialysis on the day of the study included blood pressure, and serum components. Blood pressure was monitored during dialysis and recorded upon completion of dialysis. Study variables collected at T1, T2, and T3 included salivary flow rate, pH and osmolality of the saliva, and saliva components of BUN, Cr, and the electrolytes.

#### Measures of unstimulated whole saliva (UWS)

The UWS was collected at all three time points following the guidelines developed by the School of Dentistry of the University of Southern California [21]. For each saliva collection procedure, the participants were instructed to not move their tongue or swallow (unless instructed), and to keep their eyes open. Each collection procedure consisted of requesting the participant to perform the following steps: (1) Please swallow once in order to remove excess saliva from your mouth; 92) Please hold your saliva in your mouth without swallowing for 5 min; and (at the end of 5 min) (3) Please spit your saliva into the test tube. Once the saliva collection procedure was complete, the collected saliva was stored at 4 °C for processing.

Saliva flow rate for each sample was determined by weight. The saliva was centrifuged to remove residual material or oral mucous cells (1 min @ 1000 rpm) and the upper layer was transferred to a fresh pre-weighed test tube. The solution was weighed and the volume was calculated (1 *g* = 1 mL). UWS (mL/min) was calculated by dividing the saliva volume by the collection time [11,15]. The pH, osmolality and the biochemical and electrolyte components of the saliva were then analyzed.

The pH of the saliva was determined with pH indicator strips. Salivary osmolality was measured with a freezing point depression salivary osmometer. The concentration of BUN was determined by colorimetric analysis with the absorbance set at 340/410 nm. The concentration of Cr was determined using the Jaffe alkaline picrate method with absorbance set at 505/571 nm. The concentrations of salivary sodium, potassium ions, chloride, and calcium were determined by changes in absorbance using an autoanalyzer. Absorbance was converted to concentration.

### Data collection

Data from survey questionnaires were obtained in the clinic after participants provided informed consent. Saliva collection was begun when the participant’s blood pressure had stabilized, and was conducted at three timepoints: 1 h before dialysis (T1), 1 h after dialysis had begun (the midpoint of dialysis, T2), and 1 h after completion of dialysis (T3). Blood pressure data were collected at T1 and T3.

### Statistical analysis

IBM SPSS Version 23 was used for data analysis. Descriptive statistics included mean, standard deviation (SD), and percentage. Generalized estimating equations (GEE) were used to analyze changes in UWS and the saliva variables at the three timepoints. The correlation matrix was selected to control for the effect of time [22]. Fisher’s least significant difference (LSD) was used to conduct post-hoc comparisons. A *p*-value < .05 was considered significant [23].

## Results

A total of 100 participants with ESKD agreed to participate in this study. The mean age of participants was 62.45 years (SD = 11.09; range 34–85 years); 66% were male. The mean duration of hemodialysis for 5.10 years (SD = 4.96); 67% of participants had a comorbidity of hypertension; the mean systolic blood pressure was 143.85 mm HG (SD = 18.98). Details of participants’ demographic and clinical characteristics and components of serum plasma pre-dialysis are shown in Table 1. The oral cleaning habits of the 100 participants are listed in Table 1. In terms of brushing habits, the majority of the participants (65%) brushed their teeth twice a day (once in the morning and once at night), followed by those who brushed once a day (21%); 6% of the participants did not brush their teeth or had other oral cleaning habits. For 3% of the participants, their teeth had fallen out; thus, they did not have a habit of brushing their teeth. Furthermore, 67% of the participants gargled after eating, and only 34% of the participants visited their dentist in the past year (Table 1).

**Table 1.** Demographics and baseline clinical characteristics of participants prior to receiving hemodialysis for end-stage renal disease (*N* = 100).

Variable	*n* (%)	Mean	SD
Demographics			
Age, years		62.45	11.09
≤40	3 (3%)		
41–65	57 (57%)		
≥66	40 (401%)		
Gender, male	66 (66%)		
Education			
<12 years	47 (47%)		
≧12 years	53 (53%)		
Unemployed	81 (81%)		
Married, yes	78 (78%)		
Religious, yes	90 (90%)		
Exercis*e* ≧ 3 times per week, yes	30 (30%)		
Frequency of teeth brushing in a day			
Once, after waking up in the morning 21 (21%)			
Once, before going to sleep at night 4 (4%)			
Twice, in the morning and at night 65 (65%)			
Three times or more 4 (4%)			
Do not brush teeth and other oral hygiene habits 6 (6%)			
Brush teeth for more than 3 min, yes 50 (50%)			
Gargle after meal, yes 67 (67%)			
Frequency of changing toothbrush			
One mouth 15 (15%)			
Two mouth 15 (15%)			
More than three mouths 19 (19%)			
When the bristles are splayed 46 (46%)			
Other 5 (5%)			
Brush pattern			
Bass method	1 (1%)		
Horizontally brushing	29 (29%)		
Fones method	47 (47%)		
Physiology brushing	9 (9%)		
Brush at will	3 (3%)		
Other	11 (11%)		
See a dentist in the past year, yes	34 (34%)		
Clinical characteristics			
Smoker, yes	13 (13%)		
Duration of hemodialysis (years)		5.10	4.96
Comorbidity			
Hypertension	67 (67%)		
Other	55 (55%)		
Systolic blood pressure, mm HG		143.85	18.98
Diastolic blood pressure, mm Hg		77.65	10.13
Serum components			
White blood cells, per uL		6.63	1.99
Red blood cells, per uL		3.67	0.50
Hemoglobin, g/dL		10.91	0.96
Hematocrit, %		32.55	2.83
Mean corpuscle volume		89.53	8.30
Mean corpuscular hemoglobin conc.		33.53	1.12
Platelets		171.98	62.03
Kt/v		1.48	0.17
GOT (u/L)		21.15	8.99
GPT (u/L)		18.88	12.30
Alkaline phosphatase		316.66	170.53
Albumin, g/dL		3.94	0.19
Blood urea nitrogen, mg/dL		67.99	14.35
Creatinine, mg/dL		9.63	1.68
Blood glucose (A1C), mg/dL		216.12	96.79
Electrolytes			
Na, mEq/L		136.07	3.46
K, mEq/L		4.63	0.67
Ca, mEq/L		8.98	0.92
K, mEq/L		4.61	1.19

SD: standard deviation.

Measurements of saliva flow rate, biochemical components, pH and osmolality, and electrolytes from UWS collected before and after dialysis are shown in Table 2. GEE analysis was used to examine differences between measures for the three timepoints, controlling for the effect of time. Post-hoc comparisons were conducted to examined differences among the three timepoints.

**Table 2.** Comparison of saliva variables before (pre-dialysis), during (mid-dialysis), and 1 h after completion of dialysis (post-dialysis) for participants (*N* = 100).

	Time of sampling					
	Pre-dialysis (T1)	Mid-dialysis (T2)	Post-dialysis (T3)			Comparison
Saliva variables	Mean ± SD	Mean ± SD	Mean ± SD	Wald χ^2^	*p*	Post-hoc
Saliva flow rate, ml/min	0.15 ± 0.10	0.17 ± 0.13	0.19 ± 0.12	10.40**	.006	3 > 1
Biochemicals, mg/L						
BUN	57.29 ± 38.87	32.89 ± 22.35	24.97 ± 17.94	97.12***	<.001	3 < 2 < 1
Cr	0.80 ± 0.80	0.47 ± 0.86	0.31 ± 0.45	36.98***	<.001	2 < 1; 3 < 1
pH	7.83 ± 0.75	7.63 ± 0.68	7.30 ± 0.83	30.13***	<.001	3 < 2 < 1
Osmolality	257.59 ± 280.31	247.57 ± 379.46	170.81 ± 207.74	9.71**	.008	3 < 1; 3 < 2
Electrolytes, mEq/L						
Na	40.84 ± 32.78	38.71 ± 32.47	34.59 ± 31.88	3.69	.158	
K	25.79 ± 14.48	22.23 ± 13.17	22.86 ± 11.85	6.71*	.035	2 < 1; 1 > 3
Cl	53.36 ± 26.47	45.55 ± 27.39	41.09 ± 27.56	17.64***	<.001	1 > 2; 1 > 3
Ca	4.16 ± 3.46	4.26 ± 3.76	5.50 ± 4.02	12.73**	.002	3 > 1; 3 > 2

SD: standard deviation. *p < .05, **p < .01, ***p < .001.

The mean saliva flow rate at T1 was 0.15 mL/min and flow rates increased significantly T2 and T3 compared with pre-dialysis rates (0.17 mL/min and 0.19 mL/min), respectively; Wald χ^2^ = 10. 40, *p* < .01). Post-hoc comparisons indicated the increase in saliva flow rate was only significantly higher than T1 post-dialysis (T3).

The biochemical components of BUN and Cr decreased significantly at T2 and T3 (Wald χ^2^ = 97.12 and 36.98, respectively; *p* < .001). Post-hoc comparisons showed the concentration of BUN at T3 was lower than at T2 and T2 was lower than T1, indicating a constant decrease in BUN during the course of dialysis. Post-hoc comparisons for Cr concentrations showed a significant drop from T1 to T2, and from T2 to T3, however there was no significant difference between T1 and T3.

**Figure 1.**
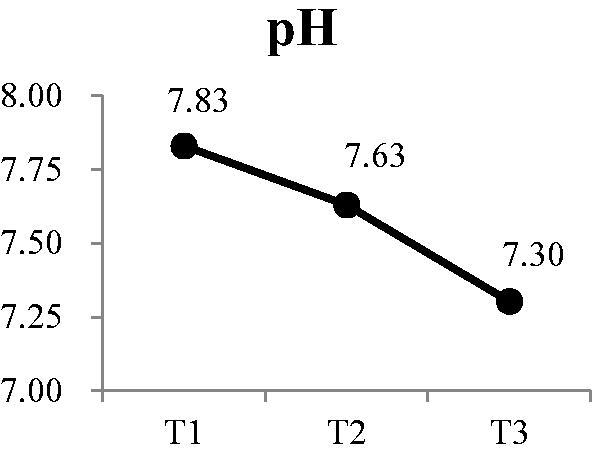
Participants’ changes in pH at each time point Participants’ changes in osmo at each time point.

**Figure 2.**
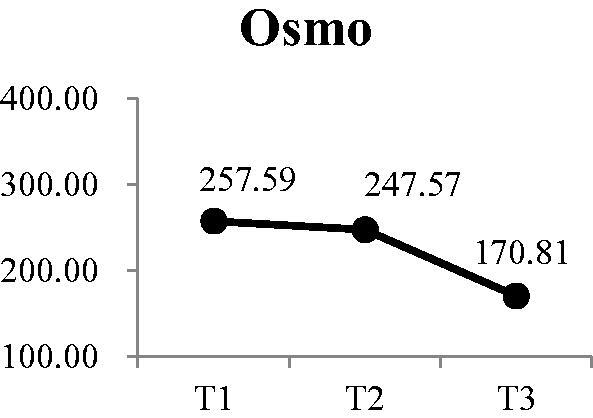
Participants’ changes in sodium at each time point Participants’ changes in potassium at each time point.

There were also significant decreases at T2 and T3 compared with T1 for pH (Figure 1) (Wald χ^2^ = 30.13, *p* < .001) and osmolality (Figure 2) (Wald χ^2^ = 9.71, *p* < .01). Post-hoc comparisons for changes in pH value indicated there was continuous decrease in the pH of saliva during dialysis. Although osmolality was lower at T2 than T1, the difference was not significant. However, osmolality at T3 was significantly lower than at T1 and T2 was significantly lower than T3

**Figure 3.**
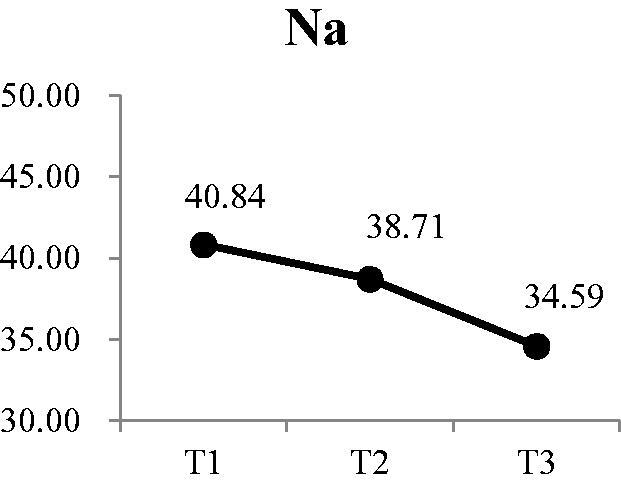
Participants’ changes in chloride at each time point Participants’ changes in calcium at each time point.

**Figure 4.**
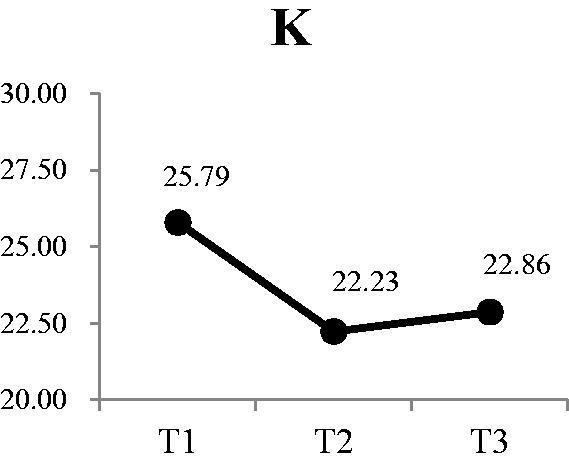
Participants’ changes in potassium at each time point.

**Figure 5.**
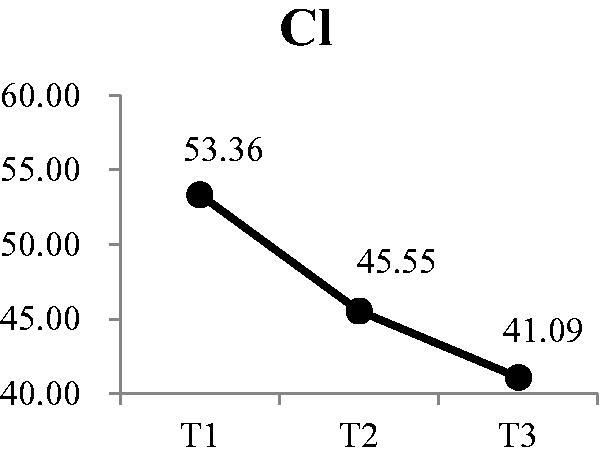
Participants’ changes in chloride at each time point.

**Figure 6.**
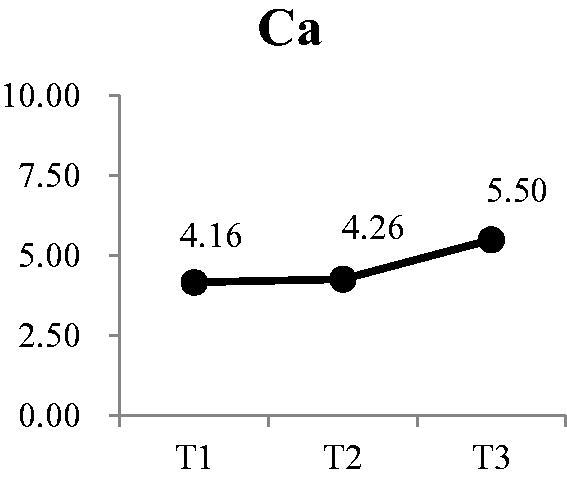
Participants’ changes in calcium at each time point.

Three of the four electrolytes in the saliva showed significant changes. The was no significant change in sodium at either T2 or T3 (Figure 3). There was a small, but significant decrease in potassium from a mean of 25.79 mEq/L at T1 to 22.86 mEq/L at T3 (Wald χ^2^ = 6.71, *p* < .05) (Figure 4). Post-hoc comparison showed both T2 and T3 were significantly lower that T1; however, there was no difference between T2 and T3. Calcium levels in the saliva increased during dialysis, from a mean of 4.16 mEq/L at T1 to 5.50 at T3 (Figure 5) (Wald χ^2^ = 12.73, *p* < .01). Post-hoc comparison showed the significant increases were T3 compared to T1 and T2, with no increase from T1 to T2. The most significant change was in the concentration of chloride (Figure 6) (Wald χ^2^ = 17.64, *p* < .001). The greatest reduction occurred from T1 (53.36 mEq/L) to T2 (45.55 mEq/L). Although the concentration at T3, was lower than T2, the difference was not significant.

Interdialytic weight gain (IDWG) is a one of factor that influences saliva flow rate. Dialysis guidelines state that ideally, IDWG should not exceed 5% of dry weight. Therefore, we divided the participants into the IDWG < 5% and ≥ 5% subgroups and compared the differences in saliva flow rate at T1, T2, and T3 (Table 3). The results revealed significant differences in the saliva flow rate between the two subgroups at T2 and T3.

**Table 3.** Differences in saliva flow rate by interdialytic weight gain (IDWG).

	IDWG/dry weight			
	<5% (*n* = 85)	≧5% (*n* = 15)		
	Mean ± SD	Mean ± SD	t	*p*
Pre-dialysis (T1)	.15	.14	.25	.80
Mid-dialysis (T2)	.18	.13	2.11	.04*
Post-dialysis (T3)	.20	.14	2.53	.02*

## Discussion

This study examined the of hemodialysis on saliva flow rate and components of saliva at by comparing unstimulated saliva collected pre-dialysis, at the midpoint of dialysis and 1 h post-dialysis. The saliva flow rate increased significantly after hemodialysis was complete, which is similar to results of other studies [15,22] in which flow rate increased during and after completion of dialysis, but was only significant between pre- and post-dialysis levels. The increase in saliva flow rate allows for an increase in removal of waste during the process of hemodialysis, which explains the lower osmolality after completion of treatment. This evidence-based information suggests the optimal time to administer an intervention to relieve dry mouth is before dialysis. Understanding a patient’s previous changes in saliva flowrate could allow nurses to administer interventions prior to dialysis according to changes in the saliva flow rate. These could include chewing gum, mouthwash, acupressure, transcutaneous electrical stimulation, or holding ice cubes in the mouth [23,24].

The mean unstimulated salivary flow rate prior to dialysis was 0.15 mL/min and reached 0.19 mL/min 1 h following completion of treatment. These flow rates were lower than previous studies with pre- and post-dialysis rates of 0.30 and .41, respectively [25], and 0.46 and 0.83, respectively [26]. The lower flowrates in our study might be due to the age of the patients in our study. The mean age for the participants in the study by Bots et al. was 54.6 years [25]. The age of participants in the study by Khanum et al. included patients as young as 18 years of age, and there was no report of the mean age for all participants [26]. Age-related changes in the function of the salivary gland contribute to reductions in unstimulated salivary flow rates [27]. Therefore, the low rates in our study may be the result of the older age of our participants.

IDWG is related to saliva flow rate; dry mouth induces thirst, which prompts increased IDWG [15,28]. The present study divided participants’ IDWG by their dry weight, multiplied the quotient by 100% to obtain their IDWG%, and then classified participants into IDWG% < 5% and IDWG% ≥ 5% subgroups. A comparison revealed that the saliva flow rate of the IDWG% < 5% subgroup was greater than that of the IDWG% ≥ 5% subgroup at T1, T2, and T3, and the differences were significant at T2 and T3. After dialysis, the saliva flow rate of the IDWG% < 5% subgroup gradually increased, testimony for ongoing significantly volume depleted state, even ate rinsing back and completion of HD session. This result may be related to the ultrafiltration rate used in the hemodialysis process. The most likely line of causality was that higher UF rate resulted (as all IDWG gained in 48 h was removed in a 2 ∼ 4 h) in more actual (intravascular) volume depletion – thus leading to less gland perfusion and saliva production. A decreased saliva flow rate stimulates thirst responses; because participants in the IDWG% ≥ 5% subgroup had low saliva flow rates, they may have consumed excessive amounts of water, which would result in increased IDWG and form a correlated cycle. Marques et al. posited that although IDWG is not related to saliva flow rate, higher IDWG implies higher risks of cardiovascular diseases and mortality [29]. Therefore, mitigating dry mouth symptoms in patients to further control water intake and IDWG is crucial. The study results can provide a reference for physicians to adjust dry weight.

The pH of the saliva gradually changed from weakly alkaline to almost neutral following completion of hemodialysis. These findings are in contrast to studies reporting hemodialysis resulted in no change in pH [26] or a decrease in pH [25]. Once again, the differences in our findings might be due to the younger age of the participants in these two studies compared with the older age of our participants. Factors such as age, sex, marital status, tobacco or soda consumption, presence of chronic diseases, tooth brushing patterns, chronic diseases, and number of missing teeth could all affect the pH value of saliva. We suggest future research should examine the relationships among saliva pH and age for patients receiving hemodialysis.

Levels of BUN, Cr, potassium, and chloride in the saliva gradually, and significantly decreased over the course of hemodialysis treatment. These changes are similar to decreases in these components when measured in blood serum. There was a significant increase in calcium concentration following dialysis. These findings provide support for a recent study by Alpdemir et al. who reported following completion of hemodialysis BUN, Cr, and potassium from saliva samples were lower than pre-dialysis levels and calcium was higher, which were similar to levels in the patients’ serum [30]. The collection of saliva is preferred to collecting blood serum for clinicians, however few researchers recognize its usefulness [30,31]. Our findings provide further support for the use of unstimulated whole saliva for measuring BUN, Cr, and electrolytes. Developing additional assays that rely on saliva could reduce the number of blood draws or finger sticks, which would reduce the number of painful, invasive procedures experienced by patients.

In spite of its strengths, our study had some limitations. First, data were collected from only one hospital in Taiwan, which might limit the generalization of our findings. Second, the research team did not take blood samples for the purpose of a correlation analysis between saliva and serum in order to minimize discomfort for our participants. Third, we might be use technology assessment to measure volume status before dialysis in the future, such as bioimpedance spectroscopy. We suggest these studies be conducted in the future in order to strengthen our findings.

In conclusion, unstimulated whole saliva was not only useful for examining changes in saliva flow rate as a physiological indicator of mouth dryness, but was also useful for examining changes in pH, osmolality, biochemical components and electrolytes over the course of hemodialysis. The results of the current study indicated that similar to blood, saliva could also serve as an indicator for predicting patients’ physiological changes throughout the process of hemodialysis. When conducting similar studies in the future, researchers could conduct blood testing simultaneously for the comparative analysis of blood and saliva. If the relationship between saliva and blood serum can be verified by more large-sample studies, saliva may be used as a physiological monitoring indicator in the future. Besides, the results of this study provide empirical evidence for medical personnel when explaining the time of interventions in health education concerning mouth dryness; thus, the effectiveness of such health education can be maximized.
